# MIMO Radar Accurate 3-D Imaging and Motion Parameter Estimation for Target with Complex Motions

**DOI:** 10.3390/s19183961

**Published:** 2019-09-13

**Authors:** Ziying Hu, Wei Wang, Fuwang Dong, Ping Huang

**Affiliations:** Automation College, Harbin Engineering University, Nantong Street 145, Harbin 150001, China; huziying@hrbeu.edu.cn (Z.H.); dongfuwang@hrbeu.edu.cn (F.D.); huangping@hrbeu.edu.cn (P.H.)

**Keywords:** MIMO radar, 3-D imaging, complex motions, high-precision, basis mismatch, atomic norm, motion parameter estimation, pairing correction

## Abstract

In this paper, three-dimensional (3-D) multiple-input multiple-output (MIMO) radar accurate localization and imaging method with motion parameter estimation is proposed for targets with complex motions. To characterize the target accurately, a multi-dimensional signal model is established including the parameters on target 3-D position, translation velocity, and rotating angular velocity. For simplicity, the signal model is transformed into three-joint two-dimensional (2-D) parametric models by analyzing the motion characteristics. Then a gridless method based on atomic norm optimization is proposed to improve precision and simultaneously avoid basis mismatch in traditional compressive sensing (CS) techniques. Once the covariance matrix is obtained by solving the corresponding semi-definite program (SDP), estimating signal parameters via rotational invariance techniques (ESPRIT) can be used to estimate the positions, then motion parameters can be obtained by Least Square (LS) method, accordingly. Afterwards, pairing correction is carried out to remove registration errors by setting judgment conditions according to resolution performance analysis, to improve the accuracy. In this way, high-precision imaging can be realized without a spectral search process, and any slight changes of target posture can be detected accurately. Simulation results show that proposed method can realize accurate localization and imaging with motion parameter estimated efficiently.

## 1. Introduction

Owing to accurate localization and imaging performance, radar is widely applied in many imaging fields. Unfortunately, the localization errors will increase so the image will be distorted and worsened when target complex motions are taken into account, particularly for slow time-varying motions containing translations and rotations [1,2].

Under this circumstance, relevant studies mostly concentrate on synthetic aperture radar (SAR), inverse synthetic aperture radar (ISAR), and 3-D interference inverse synthetic aperture radar (3-D InISAR) [3,4]. Nevertheless, the imaging accuracy and real-time performance will be worse if the synthetic aperture time cannot be optimized reasonably. Following this, some relevant improvements have been applied, containing optimization of imaging accuracy [5,6,7,8,9], selection of optimal imaging time [10], improvements of imaging efficiency by CS and sparse sampling techniques [11,12,13]. However, there are still inevitable defects. On one hand, the platform is usually required to ensure baselines unchanged during synthetic aperture time, which is impractical in actual. On the other hand, the geometric models are usually one or two-dimensional which are insufficient to accurately characterize the target, and the coordinate coincidence phenomenon will increase imaging errors when 2-D estimation results are directly expanded to 3-D [14]. In addition, the migration through resolution cell (MTRC) problem caused by the neglect of basis mismatch will further reduce the accuracy of localization and imaging.

Owing to the multi-transmitting multi-receiving mechanism, MIMO radar has bigger aperture, more array degrees of freedom and less imaging time [15,16,17], which determines it is more appropriate to realize efficient imaging. Therefore, effects of target rotations have been systematically studied with MIMO radar in relevant research. In [18], MIMO radar is proposed to solve the problems about low imaging efficiency and poor imaging quality of ISAR, but the method is limited by the computation complexity of the exhaustive search process. In [19], multi-channel Doppler computing method is proposed, and a data fitting method is used to extract motion features. However, the fitting error is large due to the approximate matching process, so the method cannot meet the requirement of high imaging precision. Based on this, literature [20] improves the estimation accuracy of micro-Doppler parameters according to a novel space geometric distribution model. For these papers, the performance of MIMO radar imaging cannot be improved fundamentally because most of them only focus on the improvements of geometric model or data fitting process, rather than analyzing and optimizing the imaging or estimation techniques in theory. In contrast, the studies in [21] greatly improve the imaging quality by combining SAR or ISAR processing techniques with MIMO radar. In this way, two radar systems can complement each other, improve the imaging efficiency, and reduce the transmitting power. Following this, Zhao et.al [22] presents a short-term shift orthogonal waveform which is more effective for parameter estimation. Then a distributed MIMO SAR/ISAR system is designed and a focusing technique is developed in [23] which can greatly improve the imaging resolution. In paper [24], the law and period of target motion are obtained by synthesizing rotation axis and comparing the target coordinates at different sampling times. Nevertheless, the synthetic aperture time is still needed, or a large number of array elements is required to refine the grids, so the real-time performance is still difficult to be guaranteed and some problems such as phase wrapping need to be solved. Moreover, the neglect of basis mismatch further increases the imaging errors and deteriorates the imaging performance. Although many current methods such as sparse adaptive calibration recovery via iterative maximum a posteriori (SACR-iMAP) method in [25] and sparsity-cognizant total least-squares (S-TLS) method in [26] can improve the imaging precision of MIMO radar by solving basis mismatch problem, the imaging errors cannot be eliminated completely.

This paper presents an accurate MIMO radar 3-D localization and imaging method with motion parameter estimation for maneuvering target. A multi-dimensional echo model is first established to precisely characterize the target, containing all the parameters of position and motion. Then it is transformed into three two-dimensional (2-D) parametric models for facilitate analysis by simplifying and analyzing specific motions features. To improve precision and eliminate the basis mismatch problem, a gridless method is presented based on atomic norm optimization. After constructing the covariance matrix by solving the SDP, parameters of motion and position can be directly calculated without spectral search process. In addition, pairing judgment and correction is carried out to remove registration errors according to resolution analysis, so that accurate imaging can be realized with precise estimation results. Finally, the simulations show that proposed method can achieve more accurate imaging with efficient estimation of motion parameters when compared to other methods.

This paper is organized as follows. In Section 2, a multi-dimensional signal model is built and transformed into joint 2-D models. In Section 3, a gridless method is introduced to achieve efficient imaging, the resolution performance is analyzed, and pairing disorders are corrected to improve accuracy. In Section 4, simulation results and discussion are given to illustrate the performance of proposed method. Finally, Section 5 gives some conclusions.

Notations: In the rest of the paper, small boldface letters denote column vectors and capital boldface letters denote matrices. ${∥\cdot ∥}_{A}$ denotes the atomic norm. T(·) denotes Toeplitz matrix. ⊗ denotes Kronecker product. ${\left(\cdot \right)}^{T}$, ${\left(\cdot \right)}^{*}$, ${\left(\cdot \right)}^{H}$, ${\left(\cdot \right)}^{-1}$ and ${\left(\cdot \right)}^{+}$ denote the transpose, conjugate, conjugate transpose, inverse, and pseudo-inverse operations, respectively.

## 2. MIMO Radar Signal Model

### 2.1. Multi-Dimensional Echo Model

**Figure 1:**
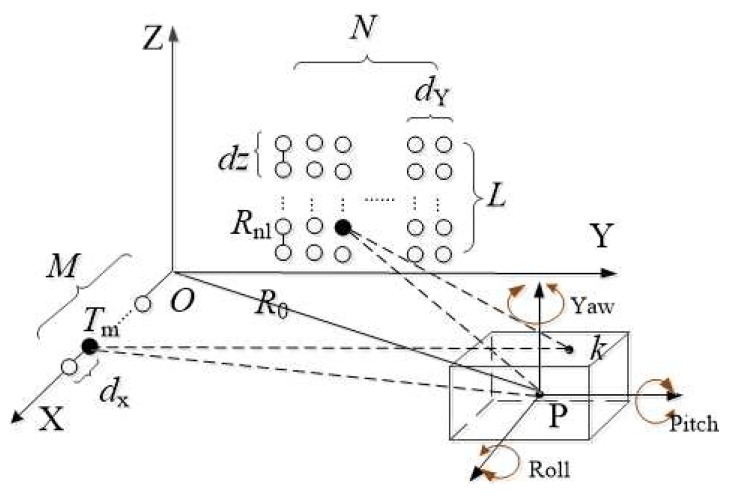
Geometry of MIMO radar array.

Appropriate array structure is necessary for MIMO radar 3-D imaging and parameter estimation [17], the layout of array in the paper is shown in Figure 1. The paper chooses a uniform linear array consists *M* transmitters along X axis direction, $T_{m}$ represents the *m-th* transmitter where m=0,1,⋯,M−1. Signal $s_{m}\left(t\right)=p_{m}\left(t\right)\exp\left(j2\pi f_{c}t+\varphi_{m}\right)$ is Hadamard orthogonal-phase encoded signal of $T_{m}$, where $p_{m}\left(t\right)$ and $f_{c}$ are envelope and carrier frequency, signal orthogonality is ensured by adjusting phase $\varphi_{m}$. Then a N×L uniform planar array is designed as the receiving array. Axial directions of Y and Z are considered to be the array line directions and $R_{nl}$ is used to represent the receiver in *n-th* row, *l-th* column where n=0,1,⋯,N−1 and l=0,1,⋯,L−1. In this paper, a large target with translations and rotations is considered in the model, thus all the scattering points have same motion states. Moreover, they all obey Swerling II distribution and scattering coefficients remain unchanged in one pulse period.

Following the array model, the echo signal at $R_{nl}$ receiver can be expressed as

(1) $$d_{n,l}\left(t\right)=\sum_{k=1}^{K}\sum_{m=0}^{M-1}\sigma_{k}\cdot \exp\left[j2\pi \left(f_{c}+f_{d}\right)\left(t-\tau_{m,n,l}^{k}\right)\right]$$

where $\sigma_{k}$ is scattering coefficient of the *k-th* scattering point, $\tau_{m,n,l}^{k}=\left(T_{m}k+R_{nl}k\right)/c$ is delay time and *c* is propagation speed of electromagnetic signal. $T_{m}k$ and $R_{nl}k$ represent the distance from $T_{m}$ to the *k-th* scattering point and the distance from *k-th* scattering point to $R_{nl}$, respectively. $f_{d}=2V_{d}/\lambda$ is Doppler frequency caused by target motions, λ is signal wavelength and $V_{d}$ is the synthesis of translation velocity and rotational angular velocity. After removing carrier, the receiving signal at $R_{nl}$ from $T_{m}$ can be written as

(2) $$d_{m,n,l}\left(t\right)=\sum_{k}^{K}\sigma_{k}\cdot \exp\left[j2\pi f_{d}t-j2\pi \left(f_{c}+f_{d}\right)\cdot \tau_{m,n,l}^{k}\right)]$$

Due to $V_{d}\ll c$, the model in (2) can be simplified as

(3) $$d_{m,n,l}\left(t\right)=\sum_{k}^{K}\sigma_{k}\cdot \exp\left(j2\pi f_{d}t\right)\cdot \exp\left[-j2\pi \left(T_{m}k+R_{nl}k\right)/\lambda \right]$$

Taking *P* as the reference center of the region, then the echo $d_{m,n,l}^{P}\left(t\right)$ reflected from *P* can be used as reference signal to compensate the target echo signal

(4) $$\begin{array}{c} D_{m,n,l}\left(t\right)=d_{m,n,l}\left(t\right)\cdot {d_{m,n,l}^{P}\left(t\right)}^{*}\\ =\sum_{k}^{K}\sigma_{k}\cdot \exp\left(j2\pi f_{d}t\right)\cdot \exp\left[-j2\pi \left(T_{m}k+R_{nl}k-T_{m}P-R_{nl}P\right)/\lambda \right] \end{array}$$

Then according to the geometrical model shown in Fig.1, taking ΔR to represent the range deviation term in (4) and it can be finally written as following, the proof is shown in Appendix A.1.

(5) $$\Delta R\approx \left[{\left(\vec{T_{m}P}\right)}^{}{}^{\prime }+{\left(\vec{R_{\mathrm{nl}}P}\right)}^{}{}^{\prime }\right]\cdot \vec{\mathrm{Pk}}$$

where ${\left(\vec{T_{m}P}\right)}^{}{}^{\prime }$ and ${\left(\vec{R_{\mathrm{nl}}P}\right)}^{}{}^{\prime }$ respectively represents the unit direction vector from $T_{m}$ and $R_{nl}$ to center *P*. Then, in order to intuitively describe the echo, we set $\left(P_{X},P_{Y},P_{Z}\right)$ and $\left(P_{X}+x_{k},P_{Y}+y_{k},P_{Z}+z_{k}\right)$ as the coordinates of *P* and point *k*, $d_{X}$ is internal spacing of transmitting array, $d_{Y}$ and $d_{Z}$ respectively denote the row and column spacing inside the receiving array. Considering the coordinates of $T_{0}$ transmitter is $\left(r_{X},0,0\right)$, the coordinates of $R_{00}$ receiver is $\left(0,r_{Y},r_{Z}\right)$, thus the coordinates of $T_{m}$ and $R_{\mathrm{nl}}$ are $\left(r_{X}+md_{X},0,0\right)$ and $\left(0,r_{Y}+nd_{Y},r_{Z}+ld_{Z}\right)$, respectively. Following this, we can get (TmP→)′≈(PX−rX−mdX,PY,PZ)(PX−rX−mdX,PY,PZ)R0R0 and (RnlP→)′≈(PX,PY−rY−ndY,PZ−rZ−ldZ)(PX,PY−rY−ndY,PZ−rZ−ldZ)R0R0, where $R_{0}$ is the reference distance from *P* to coordinate center *O*. A pulse transmitted by radar is divided into *Q* samplings, $t_{q}=q\cdot T_{p}/Q\left(q=0,1,\ldots ,Q-1\right)$ is the sampling time and $T_{p}$ is pulse width. Thus, the echo model can be written as

(6) $$D=\sum_{k=1}^{K}\sigma_{k}\cdot \left(a_{f}⊗a_{x}^{k}⊗a_{y}^{k}⊗a_{z}^{k}\right)$$

where

(7) $$\begin{array}{c} a_{f}={\left[\begin{array}{cccc} a_{f}\left(0\right) & a_{f}\left(1\right) & \cdots & a_{f}\left(Q-1\right) \end{array}\right]}^{T},\;\;\;\;a_{f}\left(q\right)=\exp\left(j2\pi f_{d}t_{q}\right)\\ a_{x}^{k}={\left[\begin{array}{cccc} a_{x}^{k}\left(0\right) & a_{x}^{k}\left(1\right) & \cdots & a_{x}^{k}\left(M-1\right) \end{array}\right]}^{T},\;\;\;a_{x}^{k}\left(m\right)=\exp\left[j2\pi \frac{2P_{X}-\left(r_{X}+md_{X}\right)}{\lambda \cdot R_{0}}\cdot x_{k}\right]\\ a_{y}^{k}={\left[\begin{array}{cccc} a_{y}^{k}\left(0\right) & a_{y}^{k}\left(1\right) & \cdots & a_{y}^{k}\left(N-1\right) \end{array}\right]}^{T},\;\;\;\;a_{y}^{k}\left(n\right)=\exp\left[j2\pi \frac{2P_{Y}-\left(r_{Y}+nd_{Y}\right)}{\lambda \cdot R_{0}}\cdot y_{k}\right]\\ a_{z}^{k}={\left[\begin{array}{cccc} a_{z}^{k}\left(0\right) & a_{z}^{k}\left(1\right) & \cdots & a_{z}^{k}\left(L-1\right) \end{array}\right]}^{T},\;\;\;\;\;a_{z}^{k}\left(l\right)=\exp\left[j2\pi \frac{2P_{Z}-\left(r_{Z}+ld_{Z}\right)}{\lambda \cdot R_{0}}\cdot z_{k}\right] \end{array}$$

However, it is difficult to directly extract parameters from the model in (6) due to the large dimension. Moreover, $V_{d}$ is also hard to be dealt because it is the synthesis of translation velocity and 3-D rotation velocity. Therefore, we transform this model into 2-D parametric models for simplicity.

### 2.2. Joint 2-D Parameter Models

Due to $f_{d}t_{q}=2V_{d}t_{q}/\lambda =2R_{q}/\lambda$, the range term produced by target motions can be expressed as following, with translation velocity *V* and 3-D rotating angular velocities $\omega_{x}$, $\omega_{y}$, $\omega_{z}$ are considered

(8) $$\begin{array}{c} \Delta R=V_{d}\cdot t_{q}\\ \Delta R_{X}=\Delta R\cdot \frac{P_{X}}{R_{0}}=\left(V\cdot t_{q}+\Delta R_{\omega }\right)\cdot \frac{P_{X}}{R_{0}}=V\cdot t_{q}\cdot \frac{P_{X}}{R_{0}}+\Delta x\\ \Delta R_{Y}=\Delta R\cdot \frac{P_{Y}}{R_{0}}=\left(V\cdot t_{q}+\Delta R_{\omega }\right)\cdot \frac{P_{Y}}{R_{0}}=V\cdot t_{q}\cdot \frac{P_{Y}}{R_{0}}+\Delta y\\ \Delta R_{Z}=\Delta R\cdot \frac{P_{Z}}{R_{0}}=\left(V\cdot t_{q}+\Delta R_{\omega }\right)\cdot \frac{P_{Z}}{R_{0}}=V\cdot t_{q}\cdot \frac{P_{Z}}{R_{0}}+\Delta z \end{array}$$

where $\Delta R_{\omega }$ is caused by target rotations and its projections are Δx, Δy and Δz, respectively. $\Delta R_{X}$, $\Delta R_{Y}$ and $\Delta R_{Z}$ are the projections of ΔR in three dimensions. Obviously, these deviation terms are only related to their own dimensions and do not affect each other.

It is noted that the velocity parameters are considered invariable due to the short processing interval of MIMO radar, i.e., *V*, $\omega_{x}$, $\omega_{y}$ and $\omega_{z}$ are all constant during *Q* samplings in one pulse. As for the roll, pitch, and yaw rotations of target shown in Figure 1, the following rotation matrices are supported by basic navigation theory

(9) $$\begin{array}{c} roll\left(\theta_{r}\left(t\right)\right)=\left[\begin{array}{ccc} 1 & 0 & 0\\ 0 & \cos\theta_{r}\left(t\right) & -\sin\theta_{r}\left(t\right)\\ 0 & \sin\theta_{r}\left(t\right) & \cos\theta_{r}\left(t\right) \end{array}\right]\\ pitch\left(\theta_{p}\left(t\right)\right)=\left[\begin{array}{ccc} \cos\theta_{p}\left(t\right) & 0 & \sin\theta_{p}\left(t\right)\\ 0 & 1 & 0\\ -\sin\theta_{p}\left(t\right) & 0 & \cos\theta_{p}\left(t\right) \end{array}\right]\\ yaw\left(\theta_{y}\left(t\right)\right)=\left[\begin{array}{ccc} \cos\theta_{y}\left(t\right) & -\sin\theta_{y}\left(t\right) & 0\\ \sin\theta_{y}\left(t\right) & \cos\theta_{y}\left(t\right) & 0\\ 0 & 0 & 1 \end{array}\right] \end{array}$$

where $\theta_{r}\left(t\right)$, $\theta_{p}\left(t\right)$ and $\theta_{y}\left(t\right)$ represent the time-varying angles caused by roll, pitch, and yaw rotations. Based on this, Δx, Δy and Δz in Equation (8) can be final expressed as following, the proof is presented in Appendix A.2.

(10) $$\begin{array}{c} \Delta x\left(t_{q}\right)=\omega_{z}\cdot y_{k}\cdot t_{q}-\omega_{y}\cdot z_{k}\cdot t_{q}\\ \Delta y\left(t_{q}\right)=\omega_{z}\cdot x_{k}\cdot t_{q}-\omega_{x}\cdot z_{k}\cdot t_{q}\\ \Delta z\left(t_{q}\right)=\omega_{y}\cdot x_{k}\cdot t_{q}-\omega_{x}\cdot y_{k}\cdot t_{q} \end{array}$$

Finally, the model in (6) can be transformed into following joint three 2-D parametric models based on Equations (8) and (10), with partial complex variables represented by new parameters $\alpha_{k}$, $\beta_{k}$, $\gamma_{k}$

(11) $$\begin{array}{c} \left\{\begin{array}{c} D_{x}=\sum_{k=1}^{K}\sigma_{k}\cdot \left(a_{x}^{k}⊗a_{\alpha }^{k}\right),\;\;\;a_{\alpha }^{k}={\left[\begin{array}{cccc} a_{\alpha }^{k}\left(0\right) & a_{\alpha }^{k}\left(1\right) & \cdots & a_{\alpha }^{k}\left(Q-1\right) \end{array}\right]}^{T}\\ D_{y}=\sum_{k=1}^{K}\sigma_{k}\cdot \left(a_{y}^{k}⊗a_{\beta }^{k}\right),\;\;\;a_{\beta }^{k}={\left[\begin{array}{cccc} a_{\beta }^{k}\left(0\right) & a_{\beta }^{k}\left(1\right) & \cdots & a_{\beta }^{k}\left(Q-1\right) \end{array}\right]}^{T}\\ D_{z}=\sum_{k=1}^{K}\sigma_{k}\cdot \left(a_{z}^{k}⊗a_{\gamma }^{k}\right),\;\;\;a_{\gamma }^{k}={\left[\begin{array}{cccc} a_{\gamma }^{k}\left(0\right) & a_{\gamma }^{k}\left(1\right) & \cdots & a_{\gamma }^{k}\left(Q-1\right) \end{array}\right]}^{T} \end{array}\right. \end{array}$$

where

(12) $$\begin{array}{c} a_{\alpha }^{k}\left(q\right)=\exp\left(j2\pi \frac{2\alpha_{k}\cdot t_{q}}{\lambda }\right),\;\;\;\alpha_{k}=\omega_{y}\cdot z_{k}-\omega_{z}\cdot y_{k}+V\cdot \frac{P_{X}}{R_{0}}\\ a_{\beta }^{k}\left(q\right)=\exp\left(j2\pi \frac{2\beta_{k}\cdot t_{q}}{\lambda }\right),\;\;\;\beta_{k}=\omega_{x}\cdot z_{k}-\omega_{z}\cdot x_{k}+V\cdot \frac{P_{Y}}{R_{0}}\\ a_{\gamma }^{k}\left(q\right)=\exp\left(j2\pi \frac{2\gamma_{k}\cdot t_{q}}{\lambda }\right),\;\;\;\gamma_{k}=\omega_{x}\cdot y_{k}-\omega_{y}\cdot x_{k}+V\cdot \frac{P_{Z}}{R_{0}} \end{array}$$

Hence, we can get the parameter models of X, Y, and Z in low-dimensional space through above decoupling process of Doppler shift term. Compared with the impossibility of estimating motion parameters directly from the Doppler frequency in (6), it becomes feasible to obtain the estimation results of target location and motion parameters from the models in (11). Simultaneously, the problem of large complexity caused by large dimension can also be solved. Then according to the consistency of three models in (11), the processing in X direction will be taken as an example.

## 3. Accurate Imaging and Motion Estimations

### 3.1. 2-D Parameters Estimation without Basis Mismatch

For radar imaging, traditional sparse recovery method such as Orthogonal Matching Pursuit (OMP) will result in basis mismatch due to the construction of sparse dictionary, which depends on the discretization process of continuous variable. In view of this, it is a feasible method to avoid this phenomenon by taking SVD decomposition of echo covariance matrix and then extracting the eigenvalues from the signal subspace accordingly. However, it should be noted that it is impractical to extract all *K*-column eigenvectors to construct the signal subspace because the echo in (11) is one-dimensional. Therefore, to solve this problem and avoid basis mismatch, a gridless method based on atomic norm optimization is proposed in this section.

As a penalty function for convex optimization problem, atomic norm shows its convenience in solving underdetermined linear inverse problems [27,28,29,30]. For X dimension echo model in (11), we define atoms

(13) $$\begin{array}{c} a\left(x,\alpha \right)=\exp\left[j2\pi \frac{2P_{X}-(r_{X}+\bar{m}d_{X})}{\lambda \cdot R_{0}}\cdot x\right]⊗\exp\left[j2\pi \frac{2\bar{q}}{\lambda }\cdot \alpha \right] \end{array}$$

where $a\left(x,\alpha \right)\in C^{MQ\times 1}$, $\bar{m}={\left[\begin{array}{cccc} 0 & 1 & \cdots & M-1 \end{array}\right]}^{T}$ and $\bar{q}={\left[\begin{array}{cccc} t_{0} & t_{1} & \cdots & t_{Q-1} \end{array}\right]}^{T}$. So, the model can be written as

(14) $$D_{x}=\sum_{k}^{K}\sigma_{k}\cdot a\left(x_{k},\alpha_{k}\right)$$

Then the atomic set is defined as $A=\{a\left(x,\alpha \right),x\in \left[x_{min},x_{max}\right],\alpha \in \left[\alpha_{min},\alpha_{max}\right]\}$ where $\left[x_{min},x_{max}\right]$ and $\left[\alpha_{min},\alpha_{max}\right]$ represent the range of *x* and α. Obviously, the basic components in A construct the full echo $D_{x}$. In this model, A and σ are considered continuous. Therefore, the atomic norm of the echo can be expressed as

(15) $${∥D_{x}∥}_{A}=\inf\left\{\sum_{k=1}^{K}\left|\sigma_{k}\right|:D_{x}=\sum_{k}^{K}\sigma_{k}\cdot a\left(x_{k},\alpha_{k}\right)\right\}$$

where $\sigma_{k}=\left|\sigma_{k}\right|e^{j\phi_{k}}$, $\sigma_{k}$ and $\phi_{k}$ are amplitude and initial phase. However, it is impractical to directly construct the covariance matrix of the echo because it is not Toeplitz. Considering this, the dimension of echo is extended: $\Lambda =E\sigma \sigma^{H}=diag\left({\left|\sigma_{1}\right|}^{2},\cdots ,{\left|\sigma_{K}\right|}^{2}\right)$, so that σ can be replaced by an equivalent diagonal matrix. Thus, we can get the rank-K matrix

(16) $$\begin{array}{c} R=ED_{x}D_{x}^{H}=A\left(x,\alpha \right)\Lambda A{\left(x,\alpha \right)}^{H}=\sum_{k}^{K}{\left|\sigma_{k}\right|}^{2}\cdot a\left(x_{k},\alpha_{k}\right)a{\left(x_{k},\alpha_{k}\right)}^{H} \end{array}$$

where $A\left(x,\alpha \right)=[a\left(x_{min},\alpha_{min}\right),\cdots ,a\left(x_{max},\alpha_{max}\right)]$ and it is obviously a Vandermonde matrix with infinite columns. Then, with Gaussian noise considered in $D_{x}$, the covariance matrix can be construct based on the optimization problem

(17) $$\hat{D}=\underset{\mu }{\arg\min}\frac{1}{2}{∥\mu -D_{x}∥}_{F}^{2}+\frac{\rho }{2}{∥\mu ∥}_{A}$$

where μ is denoised truth echo and ρ is the regularization coefficient. Accordingly, this atomic norm minimization problem can be transformed into an approximate semi-definite program (SDP) as

(18) $$\begin{array}{c} \mathrm{minimize}\frac{1}{2}{∥\mu -D_{x}∥}_{F}^{2}+\frac{\rho }{2}\left[\frac{1}{MQ}trace\left(T\left(s\right)\right)+\xi \right]\\ \mathrm{subject}\mathrm{to}\left[\begin{array}{cc} T(s) & \mu \\ \mu & \xi \end{array}\right]\geq 0 \end{array}$$

where $s=\sum_{k}^{K}{\left|\sigma_{k}\right|}^{2}\cdot a\left(x_{k},\alpha_{k}\right)$, $\xi =\sum_{k=1}^{K}{\left|\sigma_{k}\right|}^{2}$, T(s) denotes the Toeplitz covariance matrix and its first column is s. Furthermore, it has been proved that the noise can be efficiently suppressed by the optimization condition, i.e., the obtained s mainly includes the scattered sampling data even at low SNR.

According to the Toeplitz covariance matrix T(s), the estimation of parameters can be realized based on ESPRIT algorithm. K large singular values can be found and relevant signal subspace $U_{s}$ can be obtained by taking singular value decomposition of T(s), then two subspace of $U_{s}$ can be obtained as

(19) $$\begin{array}{c} U_{s_{1}}=W_{1}\cdot U_{s}\\ U_{s_{2}}=W_{2}\cdot U_{s} \end{array}$$

where $W_{1}=\left[\begin{array}{cc} I_{\left(M-1)\times (M-1\right)} & 0_{(M-1)\times 1} \end{array}\right]$ and $W_{2}=\left[\begin{array}{cc} 0_{(M-1)\times 1} & I_{\left(M-1)\times (M-1\right)} \end{array}\right]$. Following this, we can get

(20) $$\Psi =U_{s_{1}}^{+}\cdot U_{s_{2}}$$

Then the parameters *x* and α can be directly obtained according to (13) with the *K* eigenvalues extracted from Ψ by eigenvalue decomposition. Following the results, scattering coefficients distributions of the points in X-α plane can be expressed as

(21) $$\sigma_{x}={\left(A_{0}^{H}A_{0}\right)}^{-1}A_{0}^{H}D_{x}$$

where $A_{0}\in C^{MQ\times K}$ is constructed by selecting corresponding columns in A(x,α), according to the parameters extraction results $\left[\begin{array}{cccc} x_{1} & x_{2} & \cdots & x_{K} \end{array}\right]$ and $\left[\begin{array}{cccc} \alpha_{1} & \alpha_{2} & \cdots & \alpha_{K} \end{array}\right]$ from (20).

Following this, all estimation results of three models in (11) can be obtained without any spectral search process

(22) $$\begin{array}{c} F_{X}=\left[\begin{array}{cc} \begin{array}{c} x_{1}\\ x_{2}\\ \vdots \\ x_{K} \end{array} & \begin{array}{c} \alpha_{1}\\ \alpha_{2}\\ \vdots \\ \alpha_{K} \end{array}\begin{array}{c} \sigma_{x_{1}}\\ \sigma_{x_{2}}\\ \vdots \\ \sigma_{x_{K}} \end{array} \end{array}\right],F_{Y}=\left[\begin{array}{cc} \begin{array}{c} y_{1}\\ y_{2}\\ \vdots \\ y_{K} \end{array} & \begin{array}{c} \beta_{1}\\ \beta_{2}\\ \vdots \\ \beta_{K} \end{array}\begin{array}{c} \sigma_{y_{1}}\\ \sigma_{y_{2}}\\ \vdots \\ \sigma_{y_{K}} \end{array} \end{array}\right],F_{Z}=\left[\begin{array}{cc} \begin{array}{c} z_{1}\\ z_{2}\\ \vdots \\ z_{K} \end{array} & \begin{array}{c} \gamma_{1}\\ \gamma_{2}\\ \vdots \\ \gamma_{K} \end{array}\begin{array}{c} \sigma_{z_{1}}\\ \sigma_{z_{2}}\\ \vdots \\ \sigma_{z_{K}} \end{array} \end{array}\right] \end{array}$$

### 3.2. Target 3-D Imaging with Motion Parameters Estimated

According to (22), $F_{X}$, $F_{Y}$ and $F_{Z}$ only represent the distributions of scattering points in X-α, Y-β and Z-γ planes, but the orders of the points are different in these matrices. For example, the *k-th* point may locates on the *a-th* row in $F_{X}$, but the *b-th* row in $F_{Y}$ and the *c-th* row in $F_{Z}$, where *a*, *b*, *c* are different because it maybe not one-to-one with the rows among $F_{X}$, $F_{Y}$ and $F_{Z}$. So the true 3-D imaging result cannot be obtained unless the coordinates in $F_{X}$, $F_{Y}$ and $F_{Z}$ are paired accurately. Based on this, a set is constructed by combining all of X, Y and Z coordinates in (22), which surely contains the coordinates of *K* true scattering points.

(23) $$\begin{array}{c} \left\{\Gamma =\left[g_{0,0,0},\cdots ,g_{\varsigma ,\varepsilon ,\zeta },\cdots ,g_{K,K,K}\right]\left|g_{\varsigma ,\varepsilon ,\zeta }=a_{x}^{\varsigma }⊗a_{y}^{\varepsilon }⊗a_{z}^{\zeta }\right.,\right.\\ \left.\varsigma =1,2,\cdots ,K,\varepsilon =1,2,\cdots ,K,\zeta =1,2,\cdots ,K\right\} \end{array}$$

where

(24) $$\begin{array}{c} a_{x}^{\varsigma }={\left[\begin{array}{ccc} \exp(j2\pi \frac{2P_{X}-r_{X}}{\lambda \cdot R_{0}}\cdot x_{\varsigma }) & \cdots & \exp(j2\pi \frac{2P_{X}-r_{X}-\left(M-1\right)\cdot d_{X}}{\lambda \cdot R_{0}}\cdot x_{\varsigma }) \end{array}\right]}^{T}\\ a_{y}^{\varepsilon }={\left[\begin{array}{ccc} \exp(j2\pi \frac{2P_{Y}-r_{Y}}{\lambda \cdot R_{0}}\cdot y_{\varepsilon }) & \cdots & \;\;\exp(j2\pi \frac{2P_{Y}-r_{Y}-\left(N-1\right)\cdot d_{Y}}{\lambda \cdot R_{0}}\cdot y_{\varepsilon } \end{array})\right]}^{T}\\ a_{z}^{\zeta }={\left[\begin{array}{ccc} \exp(j2\pi \frac{2P_{Z}-r_{Z}}{\lambda \cdot R_{0}}\cdot z_{\zeta }) & \cdots & \;\;\exp(j2\pi \frac{2P_{Z}-r_{Z}-\left(L-1\right)\cdot d_{Z}}{\lambda \cdot R_{0}}\cdot z_{\zeta } \end{array})\right]}^{T} \end{array}$$

Then a set is defined as $\chi =[\sigma_{0,0,0},\cdots ,\sigma_{\varsigma ,\varepsilon ,\zeta },\cdots ,\sigma_{K,K,K}]$. As for the elements in $\sigma_{\varsigma ,\varepsilon ,\zeta }$, σς0,ε0,ζ0=(σxς+σyε+σzζ)σxς+σyε+σzζ)33 only if $\varsigma_{0}=\varsigma$, $\varepsilon_{0}=\varepsilon$ and $\zeta_{0}=\zeta$, otherwise $\sigma_{\varsigma_{0},\varepsilon_{0},\zeta_{0}}=0$. Then the true positions can be obtained by following optimization problem

(25) $$\min{∥D-\Gamma \cdot \sigma_{\varsigma ,\varepsilon ,\zeta }∥}_{2}^{2},\;\;\;\sigma_{\varsigma ,\varepsilon ,\zeta }\in \chi$$

where $D={\left[D_{1,1,1}\left(t_{1}\right),\cdots ,D_{M,N,L}\left(t_{1}\right)\right]}^{T}$. All the parameters are all known in this problem, so it is actually a process of finding *K* minimum values from a finite set consists of $K^{3}$ elements. Therefore, 3-D imaging result is obtained

(26) $$T=[\begin{array}{cccc} \hat{X} & \hat{Y} & \hat{Z} & \hat{\sigma } \end{array}]$$

where $T\in C^{K\times 4}$, each row of **T** denotes the 3-D positions and scattering coefficients of every scattering point. $\hat{X}=J_{1}\times X$, $\hat{Y}=J_{2}\times Y$ and $\hat{Z}=J_{3}\times Z$, where $X={\left[\begin{array}{cccc} x_{1} & x_{2} & \cdots & x_{K} \end{array}\right]}^{T}$, $Y={\left[\begin{array}{cccc} y_{1} & y_{2} & \cdots & y_{K} \end{array}\right]}^{T}$, $Z={\left[\begin{array}{cccc} z_{1} & z_{2} & \cdots & z_{K} \end{array}\right]}^{T}$, $J_{1}$, $J_{2}$, $J_{3}$ can be regarded as position selection matrices according to the results of (25).

Nevertheless, the obtained results are not the most accurate because the effects of Doppler frequency shift are ignored. For this paper, accurate motion estimation plays a significant role in position compensation and target identification. Thus, efficient estimation of the motion parameters is carried out according to the relationships among the parameters in (11) and (26), we can get

(27) $$\begin{array}{c} \varphi =\omega \cdot \Phi ,\varphi =\left[\begin{array}{ccc} {\hat{\alpha }}^{T} & {\hat{\beta }}^{T} & {\hat{\gamma }}^{T} \end{array}\right]\in C^{1\times 3K},\omega =\left[\begin{array}{cccc} \omega_{x} & \omega_{y} & \omega_{z} & V \end{array}\right]\in C^{1\times 4}\\ \Phi =\left[\begin{array}{ccc} \begin{array}{c} 0\\ {\hat{Z}}^{T}\\ -{\hat{Y}}^{T}\\ \Theta_{1} \end{array} & \begin{array}{c} {\hat{Z}}^{T}\\ 0\\ -{\hat{X}}^{T}\\ \Theta_{2} \end{array} & \begin{array}{c} {\hat{Y}}^{T}\\ -{\hat{X}}^{T}\\ 0\\ \Theta_{3} \end{array} \end{array}\right]\in C^{4\times 3K} \end{array}$$

where $\hat{\alpha }=J_{1}\alpha$, $\hat{\beta }=J_{2}\beta$, $\hat{\gamma }=J_{3}\gamma$, α, β, γ are the second column in $F_{X}$, $F_{Y}$, $F_{Z}$, and

(28) $$\begin{array}{c} \Theta_{1}=\left[\begin{array}{cccc} \frac{P_{X}}{R_{0}} & \frac{P_{X}}{R_{0}} & \cdots & \frac{P_{X}}{R_{0}} \end{array}\right]\in C^{1\times K}\\ \Theta_{2}=\left[\begin{array}{cccc} \frac{P_{Y}}{R_{0}} & \frac{P_{Y}}{R_{0}} & \cdots & \frac{P_{Y}}{R_{0}} \end{array}\right]\in C^{1\times K}\\ \Theta_{3}=\left[\begin{array}{cccc} \frac{P_{Z}}{R_{0}} & \frac{P_{Z}}{R_{0}} & \cdots & \frac{P_{Z}}{R_{0}} \end{array}\right]\in C^{1\times K} \end{array}$$

Therefore, Least Square (LS) method can be directly used to solve the problem in (27), then motion parameter vector can be estimated

(29) $$\omega =\varphi \Phi^{H}{\left(\Phi \Phi^{H}\right)}^{-1}$$

### 3.3. Pairing Correction

The estimation precision of the motion parameters is mostly determined by (27), so it must be guaranteed that the results of (22) and (26) are correct. However, the existence of model errors will lead to the failure of parameter estimation. After pairing, the coordinates of two points in one resolution cell may be disorder. For example, $\left(x_{a},y_{a},z_{a}\right)$ and $\left(x_{b},y_{b},z_{b}\right)$ are coordinates of point *a* and point *b* in same X resolution cell, with $y_{a}$, $z_{a}$ far away from $y_{b}$, $z_{b}$ and $x_{a}$ close to $x_{b}$. In this way, it is hard to distinguish them in the pairing process due to the strong coherence between them. So the final pairing result maybe $\left(x_{b},y_{a},z_{a}\right)$ or $\left(x_{a},y_{b},z_{b}\right)$, which will break the structure of $J_{1}$. As a result, the estimation of $\omega_{y}$ and $\omega_{z}$ in (29) will be inaccurate due to $\hat{\alpha }=J_{1}\alpha$ in (27). Similarly, when y or z coordinates are difficult to distinguish, the estimation of motion parameters will also be affected. Therefore, pairing correction is carried out in this section to remove this registration error and improve the accuracy of the method, particularly the estimation accuracy of motion parameters.

First, the imaging resolution performance of the model is studied by analyzing the point spread function theoretically. Here we define the point spread function of the X model as

(30) $$Psf\left(k,k_{0}\right)=\frac{1}{\kappa }\left|\langle a\left(x_{k},\alpha_{k}\right),a\left(x_{k_{0}},\alpha_{k_{0}}\right)\rangle \right|$$

where κ is a normalized parameter to ensure the maximum value of $Psf(k,k_{0})$ is 1. Following this, we take the mathematical simplification

(31) $$\begin{array}{c} Psf(k,k_{0})\\ =\frac{1}{\kappa }\left|\sum_{m=1}^{M}\sum_{q=1}^{Q}\exp[j2\pi \frac{2P_{X}-\left(r_{X}+md_{X}\right)}{\lambda \cdot R_{0}}\cdot \left(x_{k}-x_{k_{0}}\right)]\right.\left.\cdot \exp(j2\pi \frac{2T_{p}\cdot q}{\lambda \cdot Q}\left(\alpha_{k}-\alpha_{k_{0}}\right))\right|\\ =\frac{1}{\kappa }\left|\exp(j2\pi \frac{2P_{X}-r_{X}}{\lambda \cdot R_{0}}\cdot \left(x_{k}-x_{k_{0}}\right))\right.\cdot \sum_{m=1}^{M}\exp(j2\pi \frac{md_{X}}{\lambda \cdot R_{0}\cdot M}\cdot \left(x_{k}-x_{k_{0}}\right))\left.\cdot \sum_{q=1}^{Q}\exp(j2\pi \frac{2T_{p}\cdot q}{\lambda \cdot Q}\left(\alpha_{k}-\alpha_{k_{0}}\right))\right|\\ =\frac{1}{\kappa }\left|\frac{\sin(\pi \frac{Md_{X}}{\lambda \cdot R_{0}}\cdot \left(x_{k}-x_{k_{0}}\right))}{\sin(\pi \frac{d_{X}}{\lambda \cdot R_{0}}\cdot \left(x_{k}-x_{k_{0}}\right))}\cdot \frac{\sin(2\pi \frac{T_{p}}{\lambda }\left(\alpha_{k}-\alpha_{k_{0}}\right))}{\sin(2\pi \frac{T_{p}}{\lambda \cdot Q}\left(\alpha_{k}-\alpha_{k_{0}}\right))}\right|\\ \approx \sin c\left(\frac{Md_{X}}{\lambda \cdot R_{0}}\cdot \left(x_{k}-x_{k_{0}}\right)\right)\cdot \sin\left(2\frac{T_{p}}{\lambda }\left(\alpha_{k}-\alpha_{k_{0}}\right)\right) \end{array}$$

where $T_{p}=Q\times t_{q}$ is pulse width. Then the limit resolution in X-α plane is

(32) $$\rho_{x}=\frac{\lambda \cdot R_{0}}{Md_{X}}\;\;\;\;\;\;\rho_{\alpha }=\frac{\lambda }{2T_{p}}$$

that is, when the distance between two points in X direction is less than $\rho_{x}$, the main lobes of them will overlap and makes their X coordinates difficult to be distinguished. As a result, the estimation of motion parameters will be inaccurate.

In a similar way, the resolutions of Y-β plane and Z-γ plane can also be obtained

(33) $$\rho_{y}=\frac{\lambda \cdot R_{0}}{Nd_{Y}}\;\;\;\rho_{\beta }=\frac{\lambda }{2T_{p}}\;\;\;\rho_{z}=\frac{\lambda \cdot R_{0}}{Ld_{Z}}\;\;\;\rho_{\gamma }=\frac{\lambda }{2T_{p}}$$

In the following, a rough estimation is developed by judging and removing the points in the same cell. First, we need to determine whether there are points difficult to be distinguished. Taking X dimension as example, for any point *a* and point *b*, a judging condition is set as

(34) $$∥x_{a}-x_{b}∥\geq \rho_{x}$$

if it does not meet the condition, there must be more than one point in one cell, then these points are taken out from the pairing result. Therefore, it is actually a process of constantly eliminating indistinguishable target points by pairing judgment. The convergence condition of this process is that all the remaining target points satisfy the judgment condition in (34). Then, the parametric coarse estimation is developed with the target points satisfying (34)

(35) $$\varphi^{\prime }=\omega^{\prime }\cdot \Phi^{\prime }$$

where $\varphi^{\prime }\in C^{1\times K^{\prime }},\omega^{\prime }\in C^{1\times 4},\Phi^{\prime }\in C^{4\times K^{\prime }}$, $K^{\prime }$ denotes the number of the points satisfying (34).

With the process of pairing judgment and correction in (34) and (35), the coordinates between these points in same cell can be distinguished according to the coarse estimation of $\omega^{\prime }$. Then the position selection matrix $J_{1}$ can be corrected and Equations (26) and (27) are updated. Accordingly, Equation (29) will be carried out based on the corrected results. In this way, the registration error can be removed, and the high accuracy of localization and motion parameter estimation can be guaranteed.

**Figure 2:**
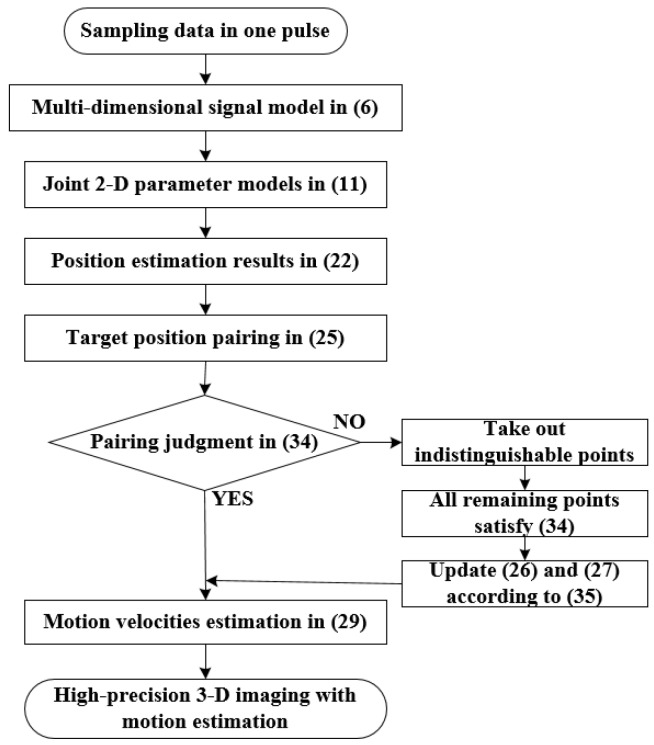
The flow chart of proposed method.

As a summary, the flow chart of the whole method is shown in Figure 2.

It is noted that the main computational load of the algorithm includes three parts. The first one is caused by the SDPT3 or ADMM [29] when the atomic norm optimization problem is solved. The second one is caused by singular value decomposition and eigenvalue decomposition when ESPRIT algorithm is used to calculate the target positions, which is $O(2\left(M^{3}+N^{3}+L^{3}\right)Q^{3}+6K^{3})$. The third one is brought by the process of pairing and motion parameter estimation, which is $O(MNLK^{3})$. Thus, the computational complexity of the algorithm is mainly affected by the number of antennas, samplings, and targets.

The method is applicable for many scenarios such as ships, airplanes, and accurate imaging can be realized with stable application environment. However, the performance will be deteriorated once the application environment becomes worse, such as the sea surface full of clutters and sea waves, which will be further studied in future research.

## 4. Simulation Results and Discussion

**Figure 3:**
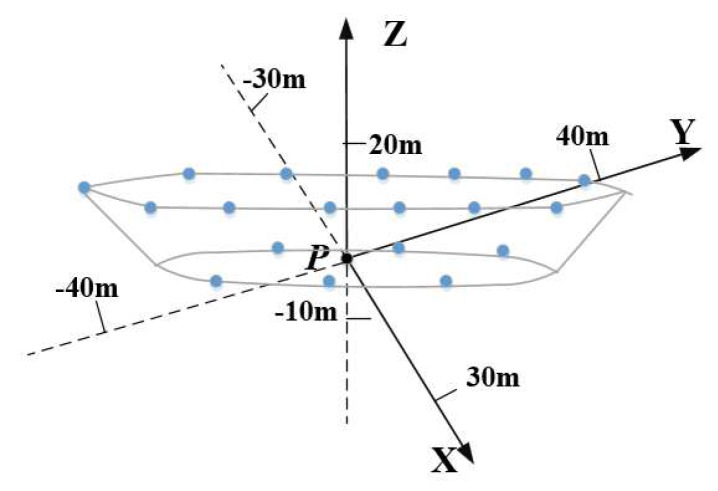
Distribution of scattering Points on the ship hull.

**Figure 4:**
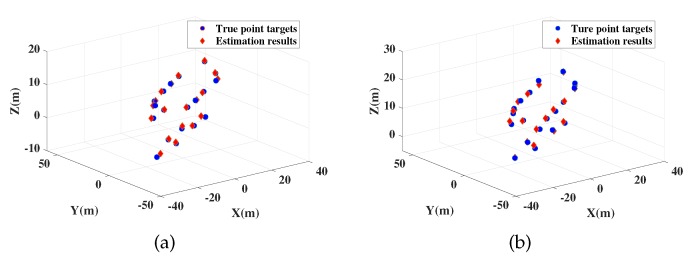
3-D MIMO Radar Imaging Results. (**a**) is imaging result at $t_{A}$; (**b**) is imaging result at $t_{B}$.

**Figure 5:**
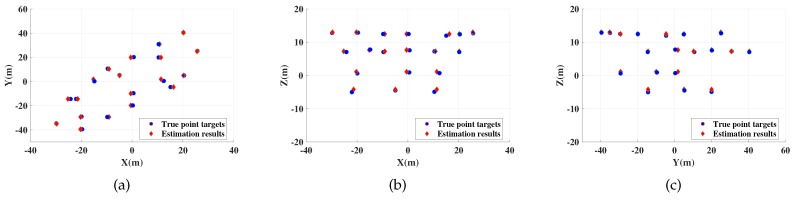
2-D projections of MIMO radar 3-D imaging results at $t_{A}$. (**a**) is projection in XY plane; (**b**) is projection in XZ plane. (**c**) is projection in YZ plane.

**Figure 6:**
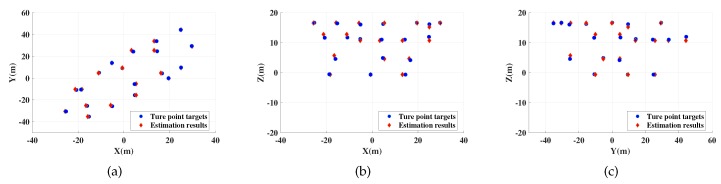
2-D projections of MIMO radar 3-D imaging results at $t_{B}$. (**a**) is projection in XY plane; (**b**) is projection in XZ plane; (**c**) is projection in YZ plane.

**Figure 7:**
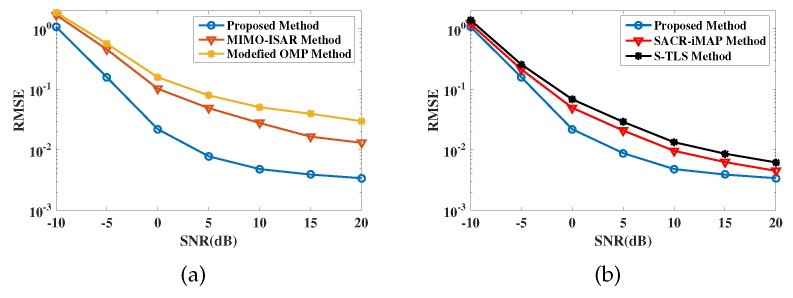
Error performance of 3-D imaging. (**a**) is the comparison with MIMO-ISAR method and modified OMP method; (**b**) is the comparison with SACR-iMAP method and S-TLS method.

**Figure 8:**
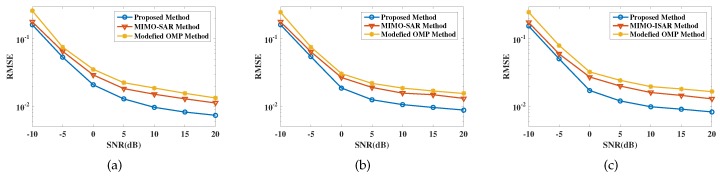
Error performance of motion estimation results. (**a**) is the comparison of roll angular velocity; (**b**) is the comparison of pitch angular velocity; (**c**) is the comparison of yaw angular velocity.

**Figure 9:**
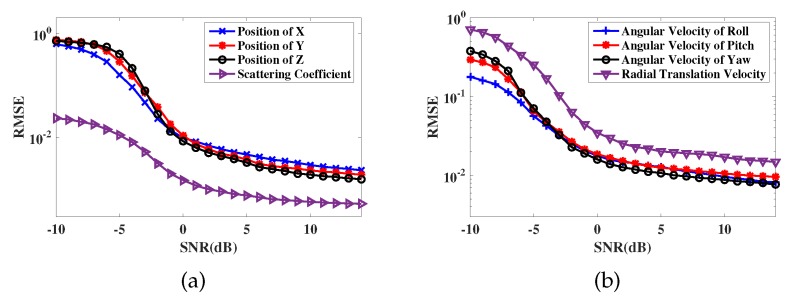
Error performance of proposed method with 500 Monte Carlo experiments. (**a**) is the error curve of 3-D localization; (**b**) is the error curve of motion parameters estimation.

In this section, relevant simulation results are shown to verify that proposed method can realize accurate imaging and motion estimation for target with complex motions. In the simulations, a ship target with radial translation and 3-D rotations is taken into account. Scattering points are set on the ship hull shown in Figure 3 and scattering coefficients are all set to 1. Following this, imaging results and motion estimation results are shown in Figure 4, Figure 5, Figure 6, Figure 7, Figure 8 and Figure 9.

First, in order to verify the feasibility of proposed method, ship target are imaged at two moments $t_{A}$ and $t_{B}$ with different motion parameters, meanwhile radar parameters are set as Table 1 and motion parameters of $t_{A}$ and $t_{B}$ are set as Table 2.

Figure 4 shows the 3-D imaging results by proposed method with SNR = 0 dB, Figure 5 and Figure 6 show their 2-D projections. In these figures, accurate localization and imaging can be intuitively presented. The deviation between true scattering points and the estimation results are small and some coincidence points appear in the figures when the estimated results of these points are more close to the real situation, which proves the feasibility and high accuracy of proposed method. Moreover, Table 3 shows the motion estimation results at $t_{A}$ and $t_{B}$. High estimation accuracy of proposed method can be verified by comparing Table 3 with Table 2, which indicates that any slight changes of target posture can be efficiently detected with the proposed imaging method.

Table 4, Table 5 and Table 6 show the time performance with different *M*, *Q*, and *L* which represent the number of transmitters, samplings, and targets, respectively. For simplicity, the effects caused by receivers are ignored because they are similar to the transmitters according to the computation analysis, so the number *N*, *L* of receivers are set as constant as shown in Table 1. Then, the rest of the parameters follow the settings in Table 1. As is shown in the tables, with the increase of *M*, *Q*, and *L*, the running time of the algorithm increases obviously. Therefore, the time efficiency decreases accordingly, which coincides with the computation analysis of the method.

Figure 7 shows the relationship between the imaging error performance and SNR. In simulation Figure 7a, we take MIMO-ISAR method [21] and modified OMP method [13] for comparison. Apparently, our method has minimum imaging errors in the figure, which verifies its high accuracy over other imaging methods. In simulation Figure 7b, the SACR-iMAP method [25] and S-TLS method [26] are simulated for references. It is obvious that these methods cannot further improve the imaging accuracy because they only focus on the improvement of 2-D resolution and the basis mismatch errors cannot be completely removed. In contrast, proposed method has higher precision. On one hand, it benefits from the denoising performance of SDP. On the other hand, target positions can be directly calculated without any spectral search process, which can avoid basis mismatch.

Figure 8 shows the comparison results of motion estimation accuracy, with MIMO-ISAR method and modified OMP method for comparison. It can be seen that proposed method has more precise motion estimation performance. In fact, the high precision is guaranteed by the gridless method and the pairing correction, where the former avoids the basis mismatch and the latter removes registration errors.

In addition, to further show the performance of proposed method under basis mismatch circumstance, Figure 9 presents the error curve of localization and motion estimation in more detail. Take 500 Monte Carlo experiments and 6 random scattering points with random motion parameters, then SNR step size is set to 1dB from −10 dB to 14 dB in the simulations. As a result, the high precision of the method can be efficiently illustrated from the simulation results Figure 9a,b.

## 5. Conclusions

In this paper, we have presented a 3-D MIMO radar imaging method with motion parameter estimation for target with complex motions. The method can reduce process difficulty by building joint 2-D parameter models. Then efficient imaging and accurate motion parameter estimation can be guaranteed by the gridless method and pairing correction process, which can eliminate basis mismatch and remove registration errors. Simulation results show that proposed method is more suitable for accurate localization and imaging of the target with complex motions.

## Figures and Tables

**Table 1:** Parameters for MIMO radar imaging.

Parameters	Values
Transmitting elements number	10
Receiving elements number	10 × 10
Internal spacing of transmitting array	3 m
Internal spacing in row and column of receiving array	4 m
Coordinate of $T_{0}$	(1 m, 0 m, 0 m)
Coordinate of $R_{00}$	(0 m, 0.5 m, 0.5 m)
Carrier frequency	35 GHz
Sampling times	30
Pulse width	600 µs
X distance	5 km
Y distance	6 km
Z distance	7 km

**Table 2:** Motion parameters for target at different times.

	Values of Motion Parameters at $t_{A}$	Values of Motion Parameters at $t_{B}$
Radial translation velocity (m/s)	4.8	8.7
Angular velocity of pitch rotation (rad/s)	0.1	0.3
Angular velocity of roll rotation (rad/s)	0.2	0.4
Angular velocity of yaw rotation (rad/s)	0.3	0.5

**Table 3:** Motion parameters estimation results.

	Estimation Results of Motion Parameters at $t_{A}$	Estimation Results of Motion Parameters at $t_{B}$
Radial translation velocity (m/s)	4.8252	8.6541
Angular velocity of pitch rotation (rad/s)	0.1136	0.2879
Angular velocity of roll rotation (rad/s)	0.2087	0.3897
Angular velocity of yaw rotation (rad/s)	0.3201	0.5221

**Table 4:** Time performance with different number of transmitters.

**The Number of Transmitters**	5	10	15	20	25	30
**Running Time (s)**	0.98	1.07	1.21	1.69	2.36	3.30

**Table 5:** Time performance with different number of samplings.

**The Number of Samplings**	5	10	15	20	25	30
**Running Time (s)**	0.84	0.96	1.17	1.50	2.19	2.98

**Table 6:** Time performance with different number of targets.

**The Number of Targets**	1	2	3	4	5	6	7	8	9	10
**Running Time (s)**	0.72	0.80	0.84	0.90	0.94	1.02	1.05	1.14	1.30	1.52

## Appendix A

### Appendix A.1. Proof of (5)

As is shown, ΔR represents the range deviation term in (4), so it can be further processed as

(A1) $$\begin{array}{c} \Delta R=\left|\vec{T_{m}k}\right|+\left|\vec{R_{\mathrm{nl}}k}\right|-\left(\left|\vec{T_{m}P}\right|+\left|\vec{R_{\mathrm{nl}}P}\right|\right)\\ =\left(\left|\vec{T_{m}k}\right|-\left|\vec{T_{m}P}\right|\right)+\left(\left|\vec{R_{\mathrm{nl}}k}\right|-\left|\vec{R_{\mathrm{nl}}P}\right|\right) \end{array}$$

In the formula, the modulus values of the vectors $\vec{T_{m}k}$, $\vec{R_{\mathrm{nl}}k}$, $\vec{T_{m}P}$ and $\vec{R_{\mathrm{nl}}P}$ are used to represent the corresponding range terms, then we can get following approximations

(A2) $$\begin{array}{c} \left|\vec{T_{m}k}\right|-\left|\vec{T_{m}P}\right|=\left|\vec{T_{m}P}+\vec{\mathrm{Pk}}\right|-\left|\vec{T_{m}P}\right|\approx {\left(\vec{T_{m}P}\right)}^{}{}^{\prime }\cdot \vec{\mathrm{Pk}}\\ \left|\vec{R_{\mathrm{nl}}k}\right|-\left|\vec{R_{\mathrm{nl}}P}\right|=\left|\vec{R_{\mathrm{nl}}P}+\vec{\mathrm{Pk}}\right|-\left|\vec{R_{\mathrm{nl}}P}\right|\approx {\left(\vec{R_{\mathrm{nl}}P}\right)}^{}{}^{\prime }\cdot \vec{\mathrm{Pk}} \end{array}$$

where ${\left(\vec{T_{m}P}\right)}^{}{}^{\prime }$ and ${\left(\vec{R_{\mathrm{nl}}P}\right)}^{}{}^{\prime }$ respectively represents the unit direction vector from $T_{m}$ and $R_{nl}$ to center *P*, then Equation (A1) can be expressed as

(A3) $$\Delta R\approx \left[{\left(\vec{T_{m}P}\right)}^{}{}^{\prime }+{\left(\vec{R_{\mathrm{nl}}P}\right)}^{}{}^{\prime }\right]\cdot \vec{\mathrm{Pk}}$$

### Appendix A.2. Proof of (10)

According to the rotation matrices, the time-varying characteristics of Δx, Δy and Δz in Equation (8) can be expressed as

(A4) $$\begin{array}{c} \left[\begin{array}{c} \Delta x(t)\\ \Delta y(t)\\ \Delta z(t) \end{array}\right]=\Omega \cdot \left[\begin{array}{c} x_{k}\\ y_{k}\\ z_{k} \end{array}\right] \end{array}$$

where $\Omega =roll\left(\theta_{r}\left(t\right)\right)\cdot pitch\left(\theta_{p}\left(t\right)\right)\cdot yaw\left(\theta_{y}\left(t\right)\right)-I_{3\times 3}$, so that we can obtain the processing results

(A5) $$\begin{array}{c} x_{k}+\Delta x\left(t_{q}\right)=x_{k}\cdot \cos\theta_{p}\left(t_{q}\right)\cdot \cos\theta_{y}\left(t_{q}\right)+y_{k}\cdot \cos\theta_{p}\left(t_{q}\right)\cdot \sin\theta_{y}\left(t_{q}\right)+z_{k}\cdot \sin\theta_{p}\left(t_{q}\right)\\ y_{k}+\Delta y\left(t_{q}\right)=y_{k}\cdot \cos\theta_{y}\left(t_{q}\right)\cdot \cos\theta_{r}\left(t_{q}\right)+z_{k}\cdot \cos\theta_{y}\left(t_{q}\right)\cdot \sin\theta_{r}\left(t_{q}\right)+x_{k}\cdot \sin\theta_{y}\left(t_{q}\right)\\ z_{k}+\Delta z\left(t_{q}\right)=z_{k}\cdot \cos\theta_{r}\left(t_{q}\right)\cdot \cos\theta_{p}\left(t_{q}\right)+x_{k}\cdot \cos\theta_{r}\left(t_{q}\right)\cdot \sin\theta_{p}\left(t_{q}\right)+y_{k}\cdot \sin\theta_{r}\left(t_{q}\right) \end{array}$$

Because rotational velocities are invariable due to the short processing interval of MIMO radar, i.e., $\omega_{x}$, $\omega_{y}$, $\omega_{z}$ are all constant during *Q* samplings in one pulse. Following this, the instantaneous angle is $\theta_{i}\left(t_{q}\right)=\omega_{i}\cdot t_{q}\left(i=r,p,y\right)$. Moreover, it is supposed that $\sin\theta_{i}\left(t_{q}\right)\approx \theta_{i}\left(t_{q}\right)=\omega_{i}\cdot t_{q}$ and cosθi(tq)≈1−θi2(tq)θi2(tq)22=1−ωi2·tq2ωi2·tq222, then we can get the following results with higher-order terms ignored.

(A6) $$\begin{array}{c} \Delta x\left(t_{q}\right)=\omega_{z}\cdot y_{k}\cdot t_{q}-\omega_{y}\cdot z_{k}\cdot t_{q}\\ \Delta y\left(t_{q}\right)=\omega_{z}\cdot x_{k}\cdot t_{q}-\omega_{x}\cdot z_{k}\cdot t_{q}\\ \Delta z\left(t_{q}\right)=\omega_{y}\cdot x_{k}\cdot t_{q}-\omega_{x}\cdot y_{k}\cdot t_{q} \end{array}$$
